# Supplementary material for: Transformation of Tetracycline by Manganese Peroxidase from Phanerochaete chrysosporium
Source: Molecules. 2021 Nov 11;26(22):6803. doi: 10.3390/molecules26226803 (PMC8619068; doi:10.3390/molecules26226803)
Supplement: Supplementary file 1 [file molecules-26-06803-s001.zip › molecules-1452286-supplementary.pdf]

Supplementary Material

S1. Materials and Methods

S1.1. Culture medium

Potato dextrose liquid, 200 g L^{-1} potato extract and 20 g L^{-1} glucose, adjusted the pH to 4.5.

Potato dextrose agar (PDA), 200 g L^{-1} potato extract, 20 g L^{-1} glucose and 20 g L^{-1} agar, adjusted the pH to 4.5.

Liquid enzyme production medium, the optimized concentrations of 10 g·L^{-1} glucose, 0.2 g·L^{-1} ammonium tartrate and 1 mmol·L^{-1} MnSO4 were used, 2.0 g KH_2PO_4, 0.1 g $CaCl_2 \cdot 2H_2O$, 0.5 g $MgSO_4 \cdot 7H_2O$, 1.0 g Tween80, 1.5 mL veratryl alcohol, 1 mg vitamin B1, 0.2 g yeast extract, 70 mL trace-element solution (1.5 g nitrilotriacetate, 1.46 g $MgSO_4 \cdot 7H_2O$, 1.0 g NaCl, 0.1 g $FeSO_4 \cdot 7H_2O$, 0.1 g $CoSO_4$, 0.1 g $ZnSO_4 \cdot 7H_2O$, 0.01 g $CuSO_4$, 0.01 g $AlK(SO_4) \cdot 12H_2O$, 0.01 g H_2BO_3, 0.01 g $NaMoO_4 \cdot 2H_2O$, 1 L water), 1L water (Tien and Kirk, 1988).

S1.2. Enzyme Activity Assay

The enzyme activity of MnP was calculated by equation S1

$$\text{MnP activity (U L}^{-1}) = \frac{\Delta A_{238} \times v_0}{\xi \times v} \times n \qquad \text{(S1)}$$

ΔA_{238} is the absorbance change value of sample per minute, v_0 is the total volume of the reaction system (1 mL), v is the solution volume of MnP (0.1 mL), ξ is the coefficient (6, 500 L $mol^{-1} \cdot cm^{-1}$), n is the dilution rate.

S1.3. Modeling

The transformation kinetics of tetracycline by MnP was calculated by the

Michaelis-Menten model (S2).

$$v = \frac{v_{max} \times S}{K_m + S} \qquad \text{(S2)}$$

v is the initial transformation rate (mg L^{-1} min^{-1}), v_{max} is the maximum initial transformation rate (mg L^{-1} min^{-1}), K_m (mg L^{-1}) is half saturation constant, and S is the initial concentration of tetracycline (mg L^{-1}). Non-linear regression analysis was conducted using the software Origin 9.0.

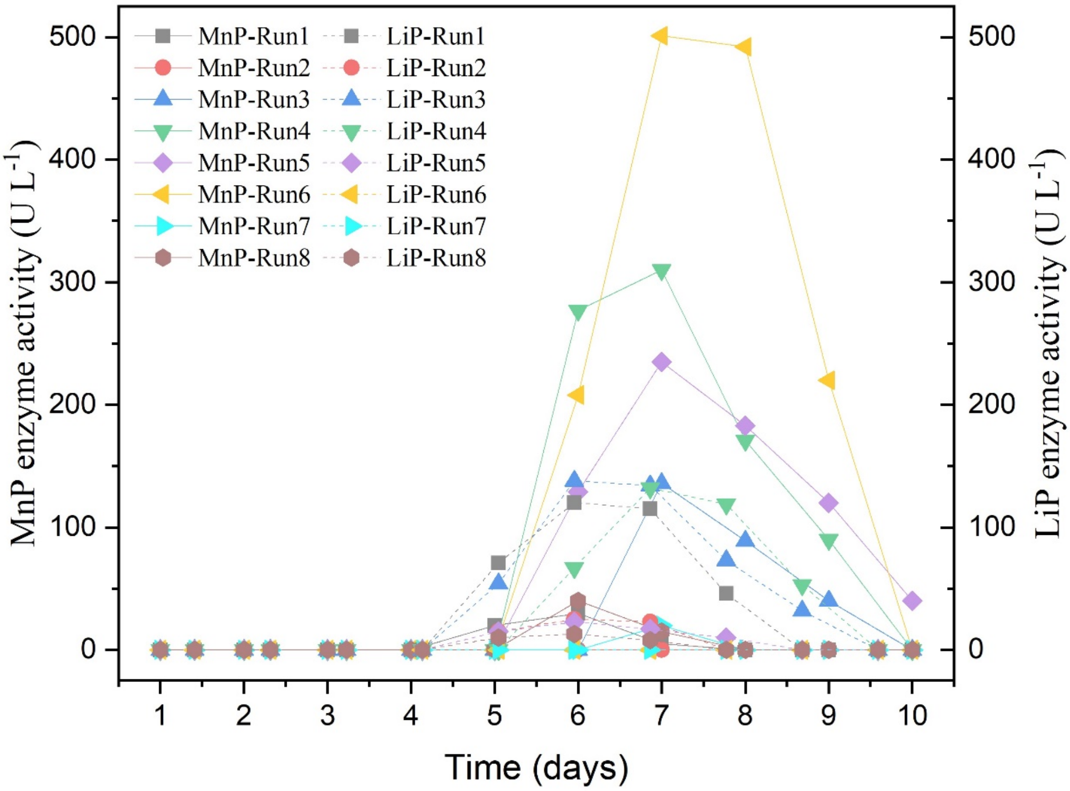

Figure S1. Expression of MnP and LiP in different runs of orthogonal experiment.

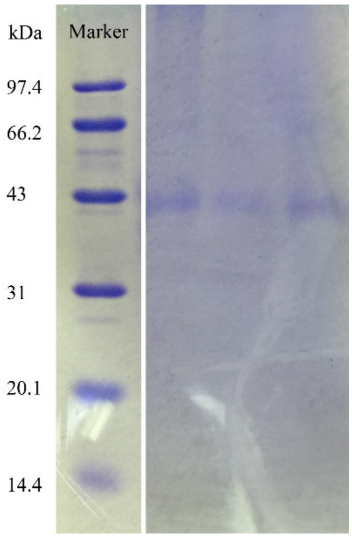

Figure S2. Electrophoretic analysis (SDS-PAGE) of purified MnP, which were stained with coomassie blue.

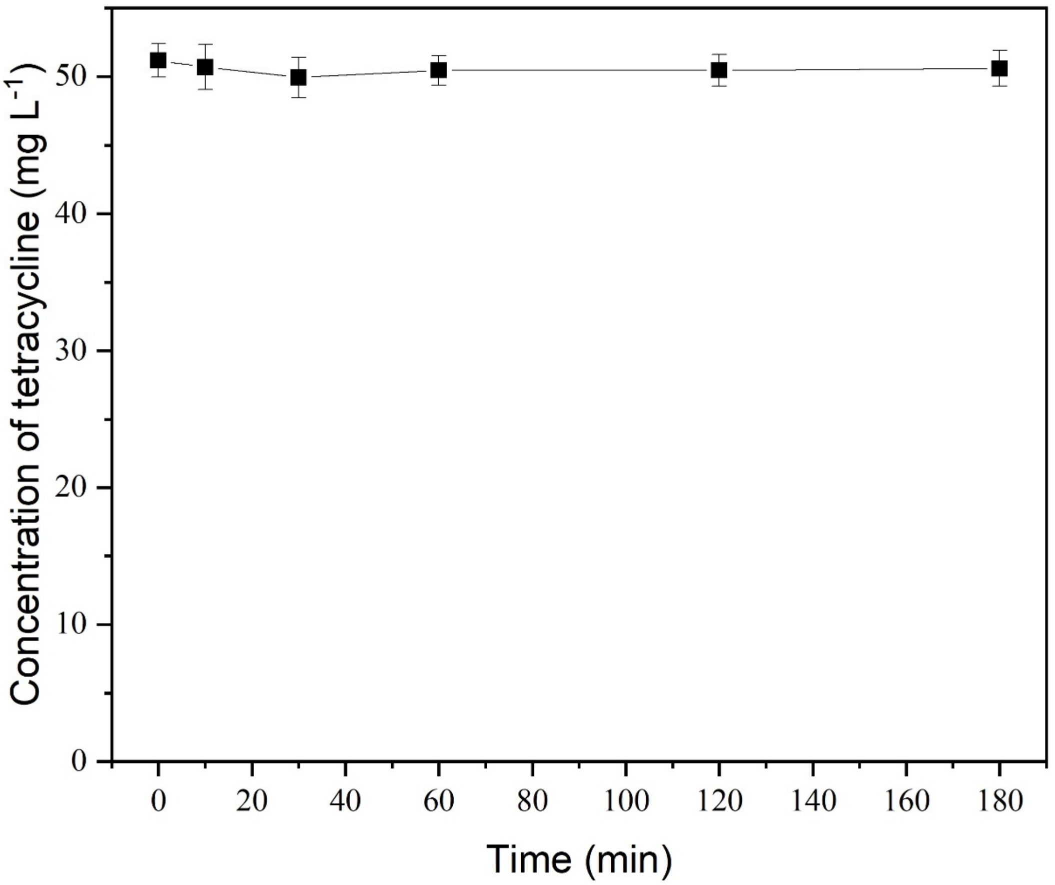

Figure S3. Treatment of tetracycline with H_2O_2 alone.

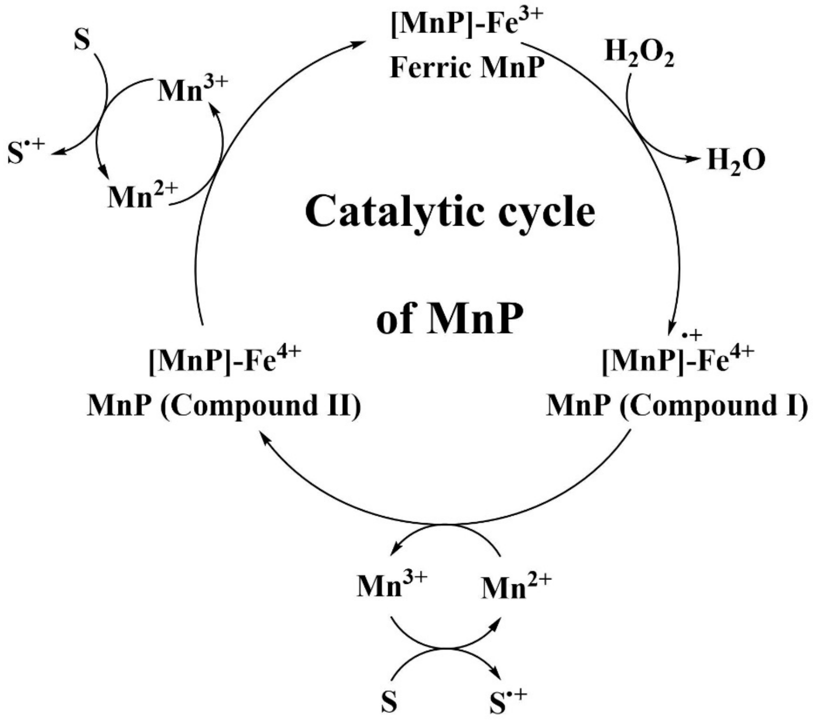

Figure S4. Mechanism of the catalytic cycle of MnP (Rahul et al., 2017).

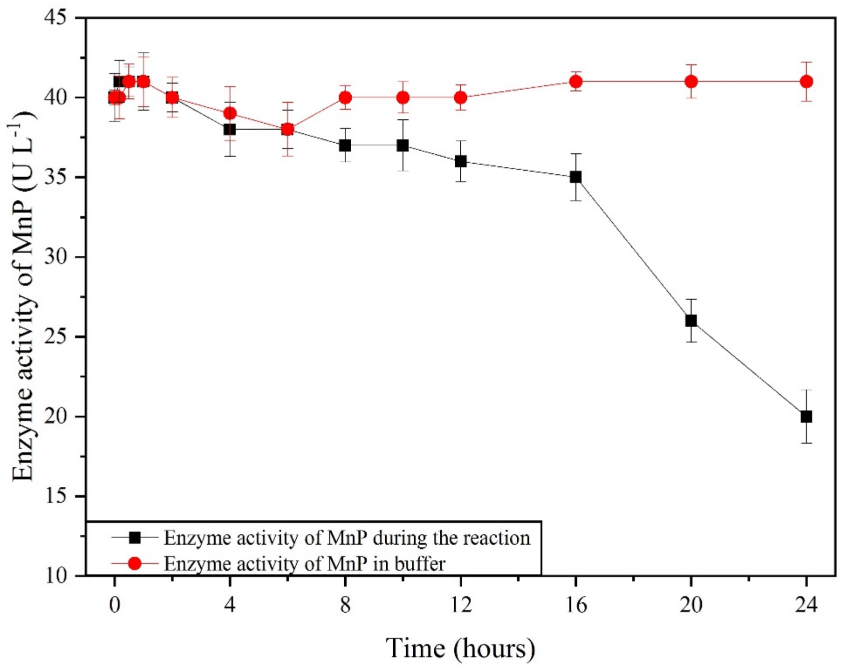

Figure S5. MnP enzyme activity changes in the catalytic system and buffer.

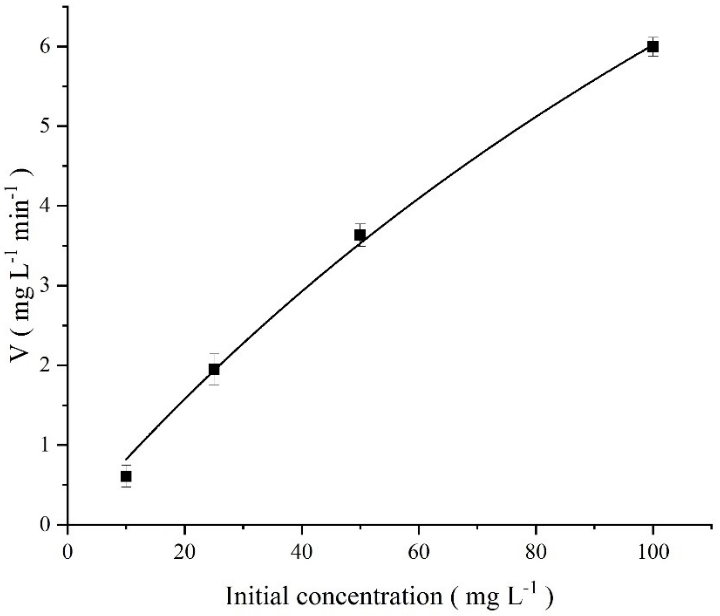

Figure S6. Simulation of transformation kinetics of tetracycline by Michaelis-Menten model in MnP system. The value of R^2 is 0.9953.

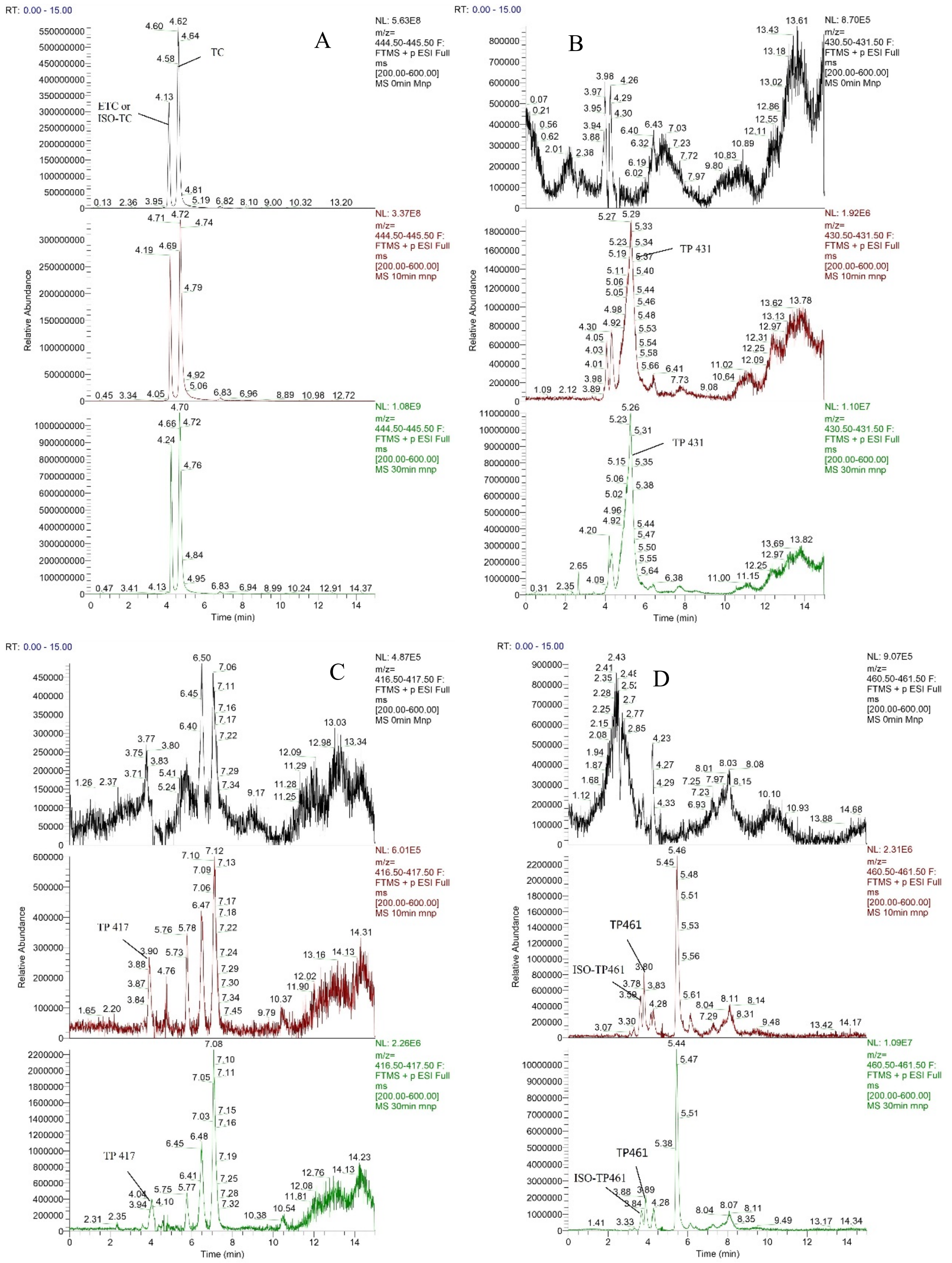

A
B
C
D
RT: 0.00 - 15.00
NL: 5.63E8 m/z= 444.50-445.50 F: FTMS + p ESI Full ms [200.00-600.00] MS 0min Mnp
NL: 3.37E8 m/z= 444.50-445.50 F: FTMS + p ESI Full ms [200.00-600.00] MS 10min mnp
NL: 1.08E9 m/z= 444.50-445.50 F: FTMS + p ESI Full ms [200.00-600.00] MS 30min mnp
TC
ETC or ISO-TC
NL: 8.70E5 m/z= 430.50-431.50 F: FTMS + p ESI Full ms [200.00-600.00] MS 0min Mnp
NL: 1.92E6 m/z= 430.50-431.50 F: FTMS + p ESI Full ms [200.00-600.00] MS 10min mnp
NL: 1.10E7 m/z= 430.50-431.50 F: FTMS + p ESI Full ms [200.00-600.00] MS 30min mnp
TP 431
NL: 4.87E5 m/z= 416.50-417.50 F: FTMS + p ESI Full ms [200.00-600.00] MS 0min Mnp
NL: 6.01E5 m/z= 416.50-417.50 F: FTMS + p ESI Full ms [200.00-600.00] MS 10min mnp
NL: 2.26E6 m/z= 416.50-417.50 F: FTMS + p ESI Full ms [200.00-600.00] MS 30min mnp
TP 417
NL: 9.07E5 m/z= 460.50-461.50 F: FTMS + p ESI Full ms [200.00-600.00] MS 0min Mnp
NL: 2.31E6 m/z= 460.50-461.50 F: FTMS + p ESI Full ms [200.00-600.00] MS 10min mnp
NL: 1.09E7 m/z= 460.50-461.50 F: FTMS + p ESI Full ms [200.00-600.00] MS 30min mnp
TP461
ISO-TP461
Relative Abundance
Time (min)

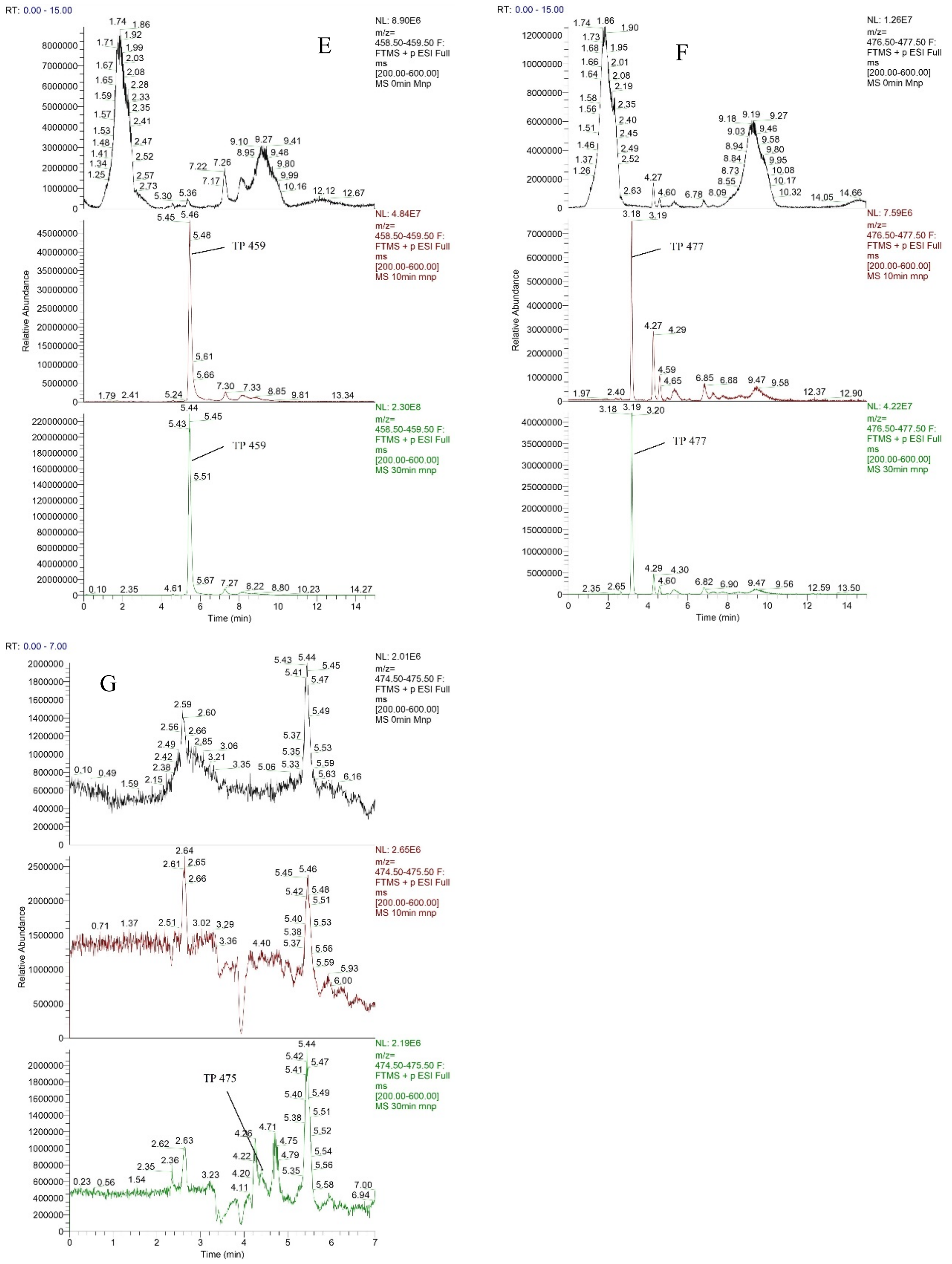

Figure S7. Extracted ion chromatograms at M/Z 445 (A), M/Z 431 (B), M/Z 417 (C), M/Z 461 (D), M/Z 459 (E), M/Z 477 (F) and M/Z 475 (G). Within each panel, the ion chromatograms from top to bottom represent the initial tetracycline parent compound sampled after 0 min, 10 min, and 30 min. The peaks were identified using mass spectrometry

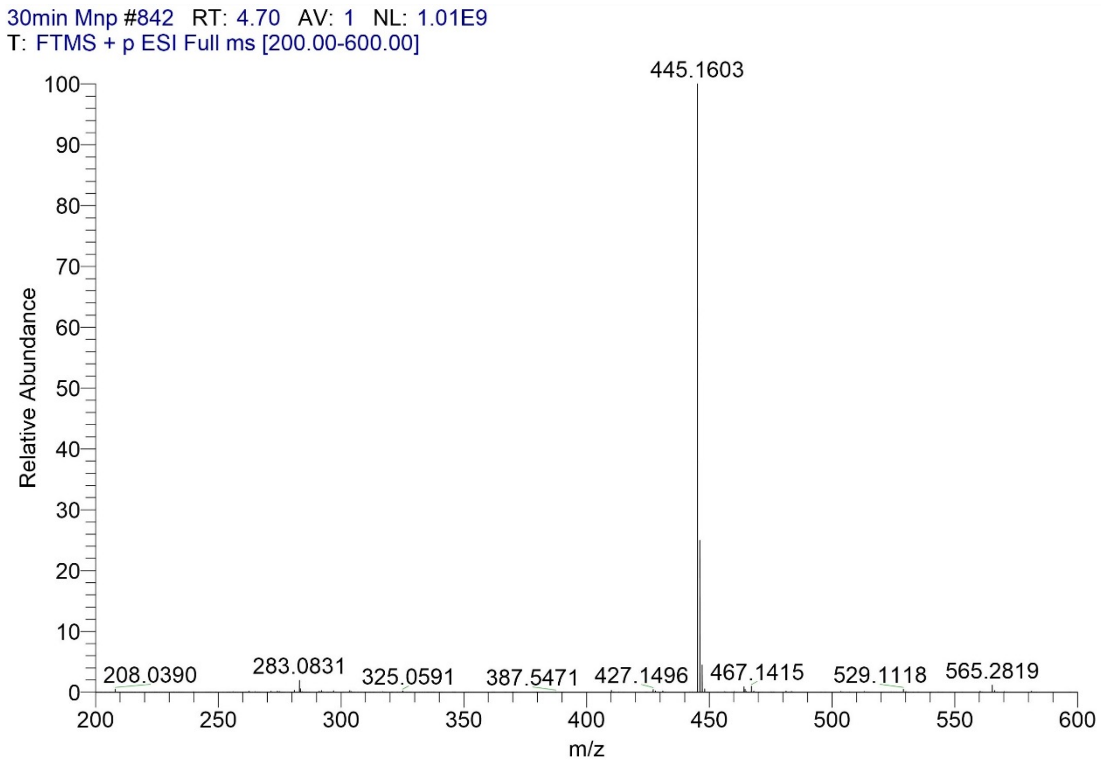

Figure S8. Mass spectrum of tetracycline.

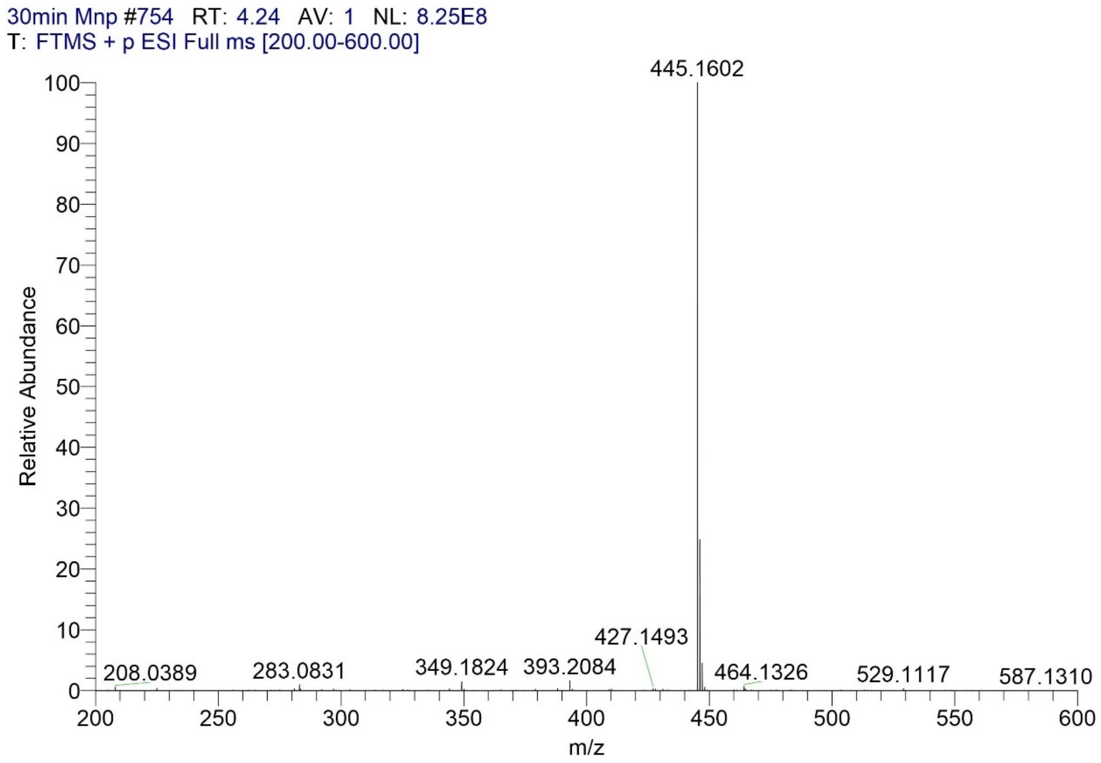

Figure S9. Mass spectrum of ETC or ISO-TC.

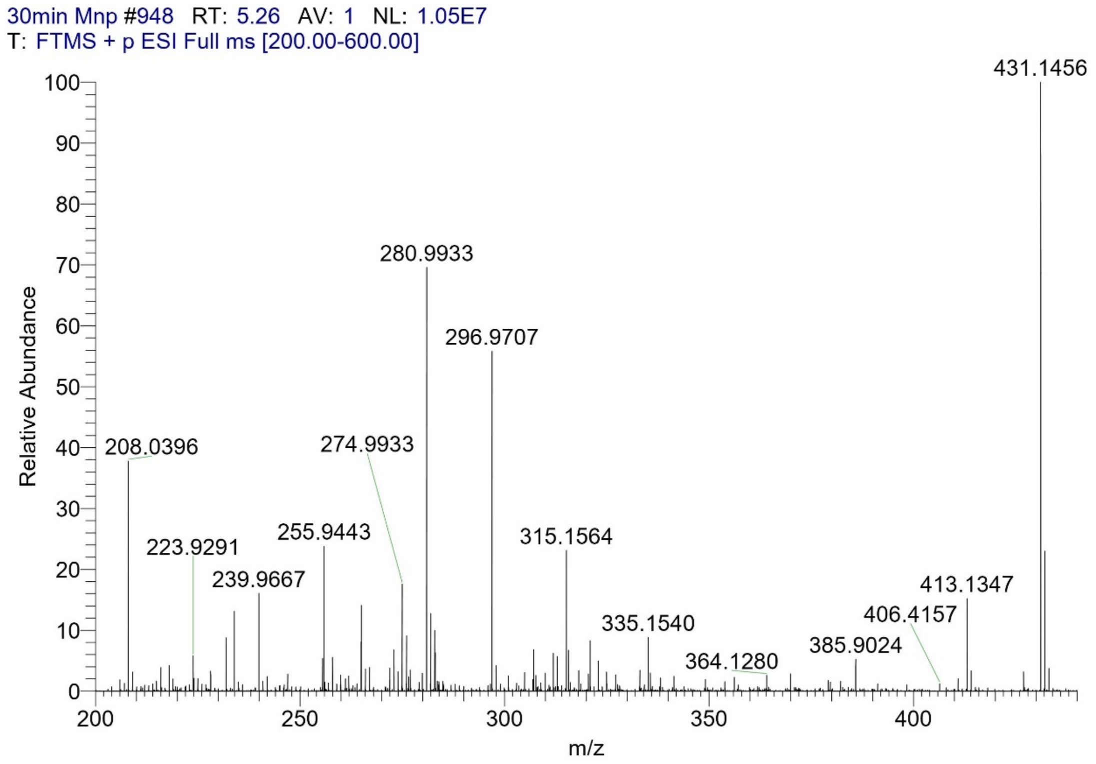

Figure S10. Mass spectrum of TP431.

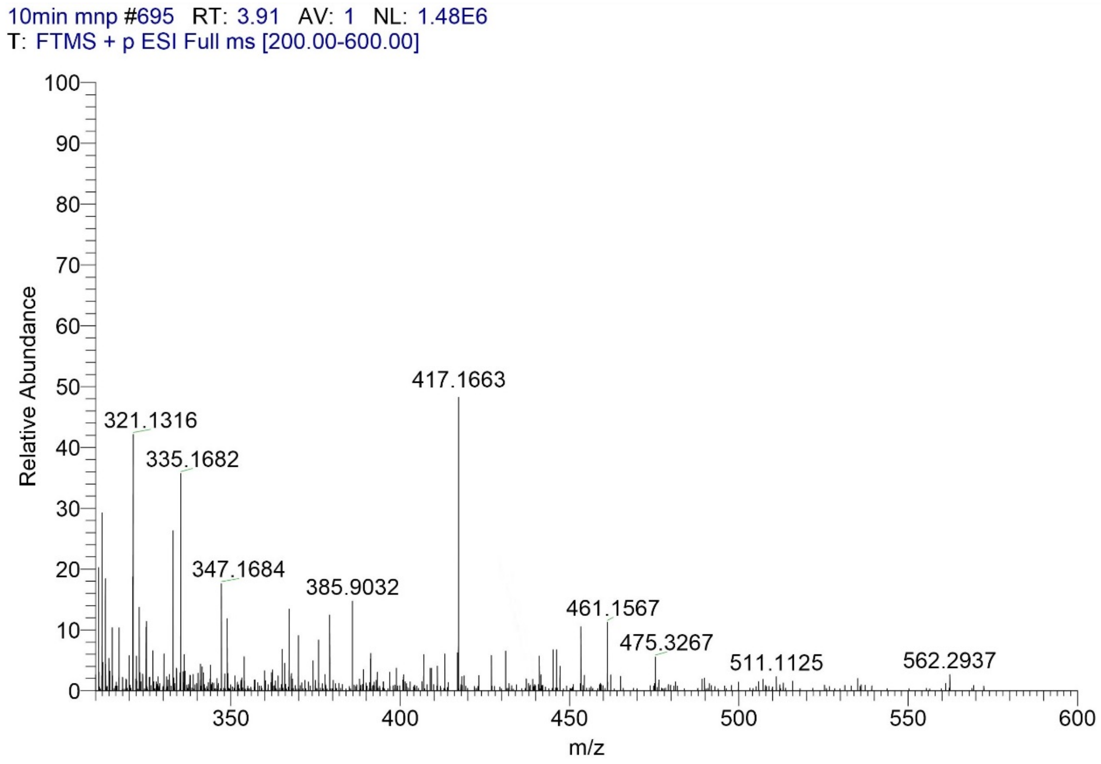

Figure S11. Mass spectrum of TP417.

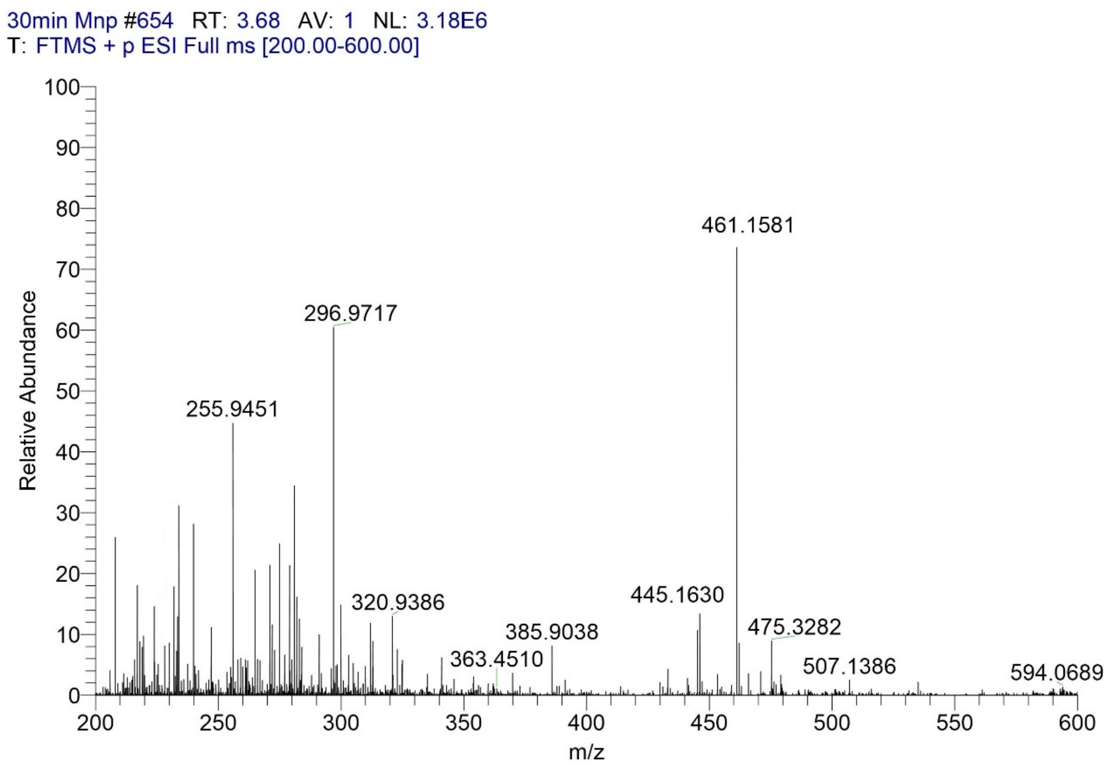

Figure S12. Mass spectrum of TP461.

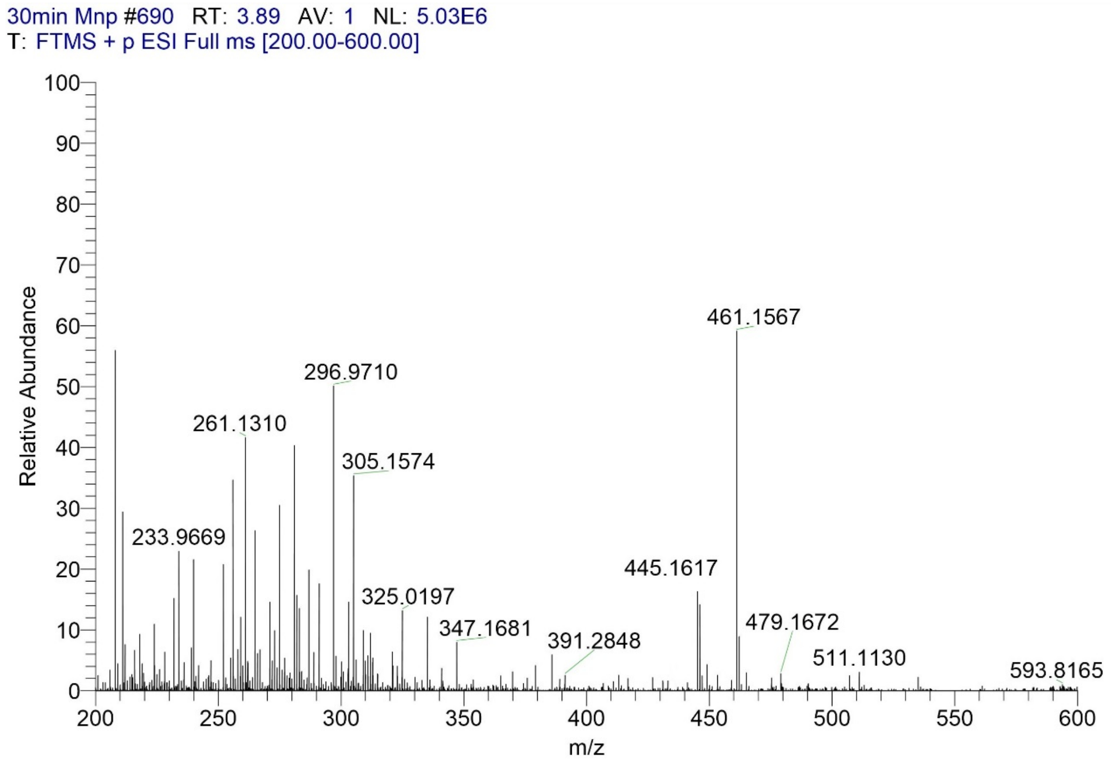

Figure S13. Mass spectrum of ISO-TP461.

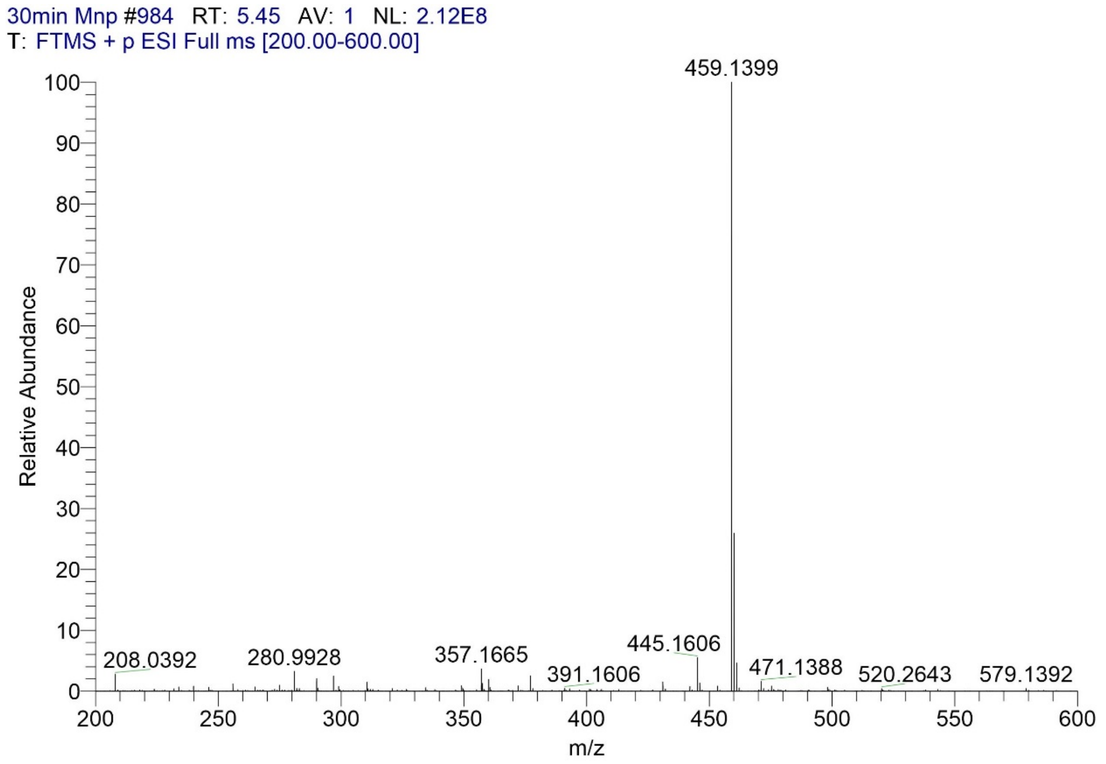

Figure S14. Mass spectrum of TP459.

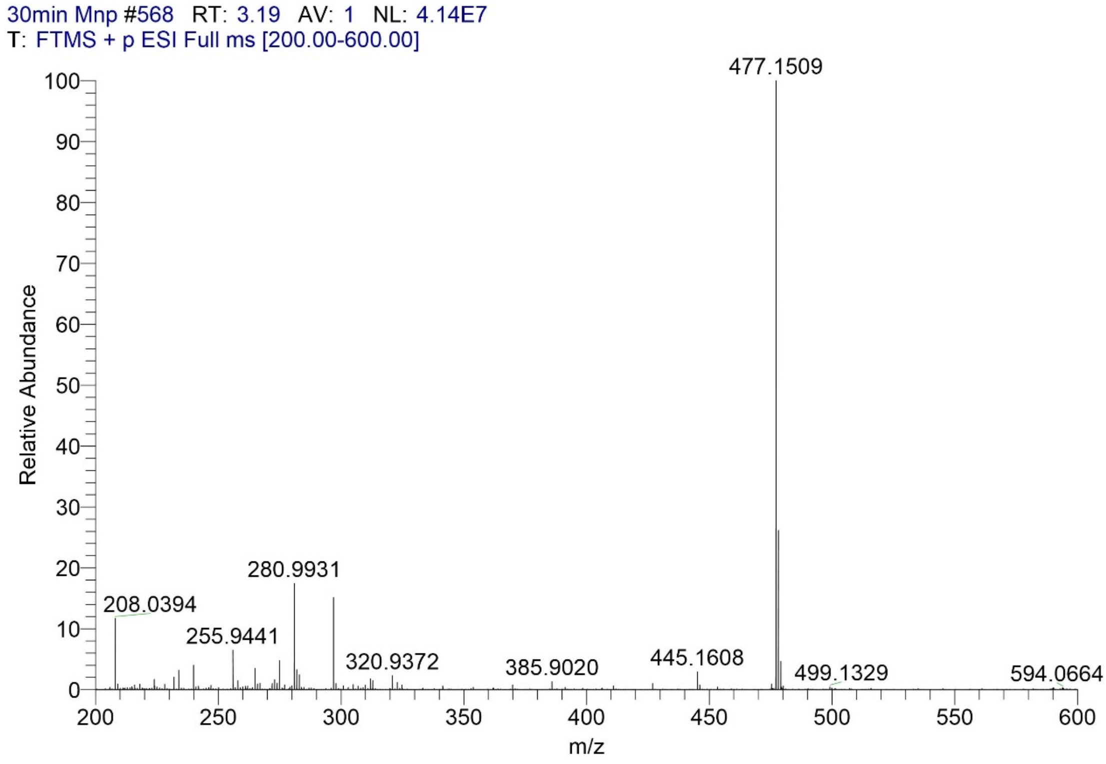

Figure S15. Mass spectrum of TP477.

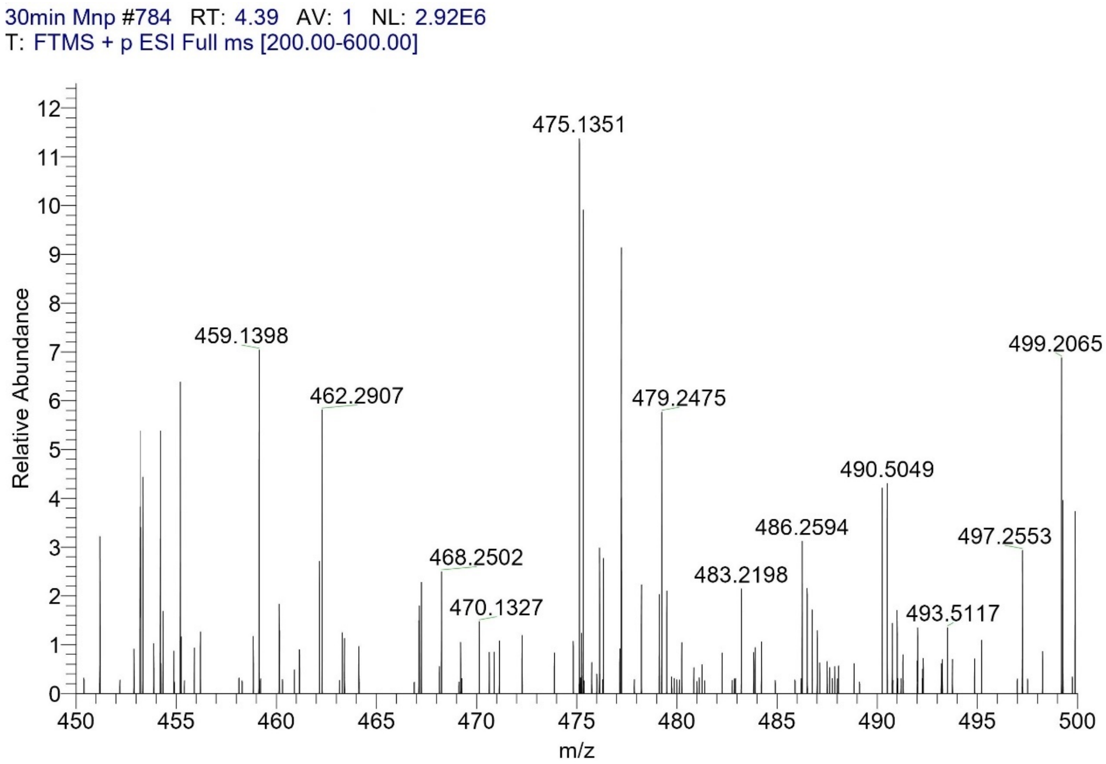

Figure S16. Mass spectrum of TP475.

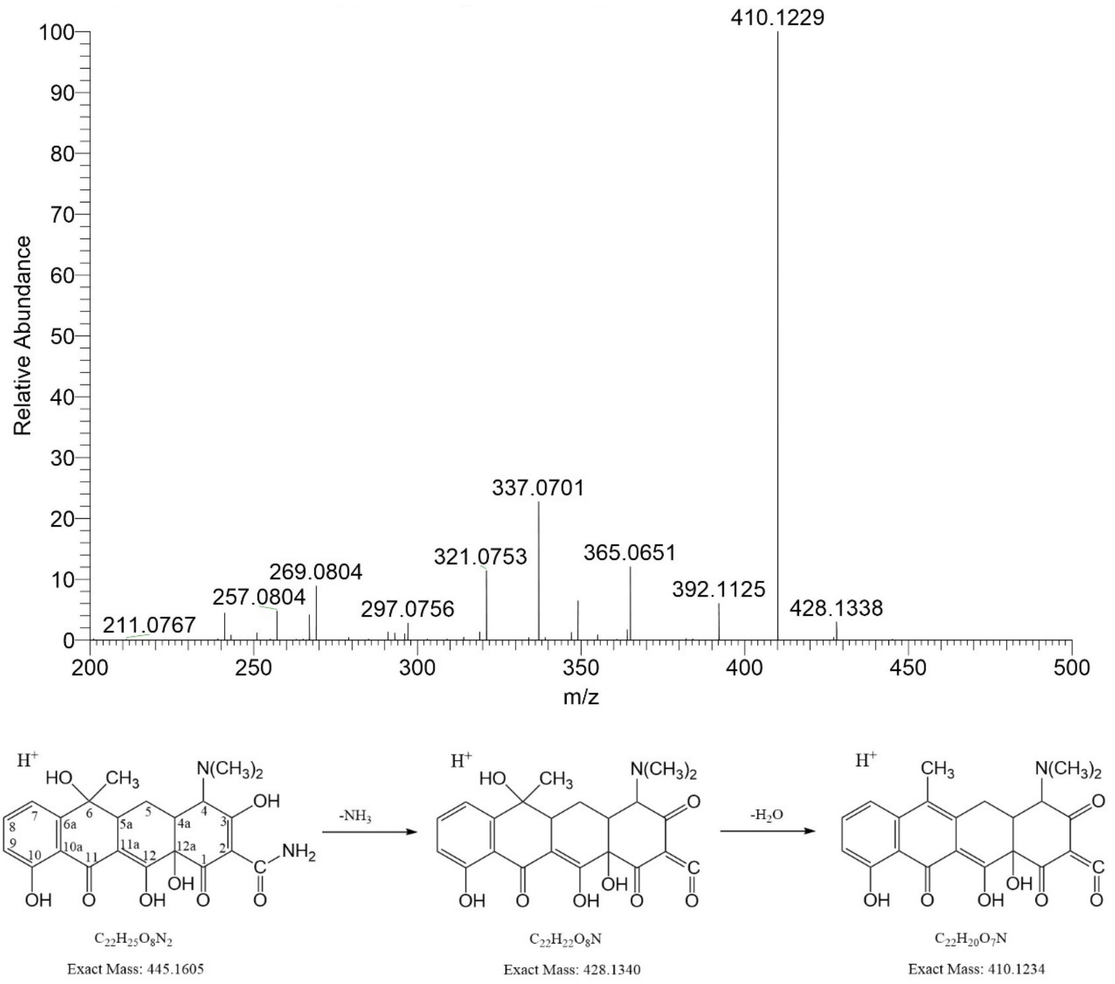

Figure S17. The MS2 fragmentation profile and proposed fragmentation pattern of tetracycline.

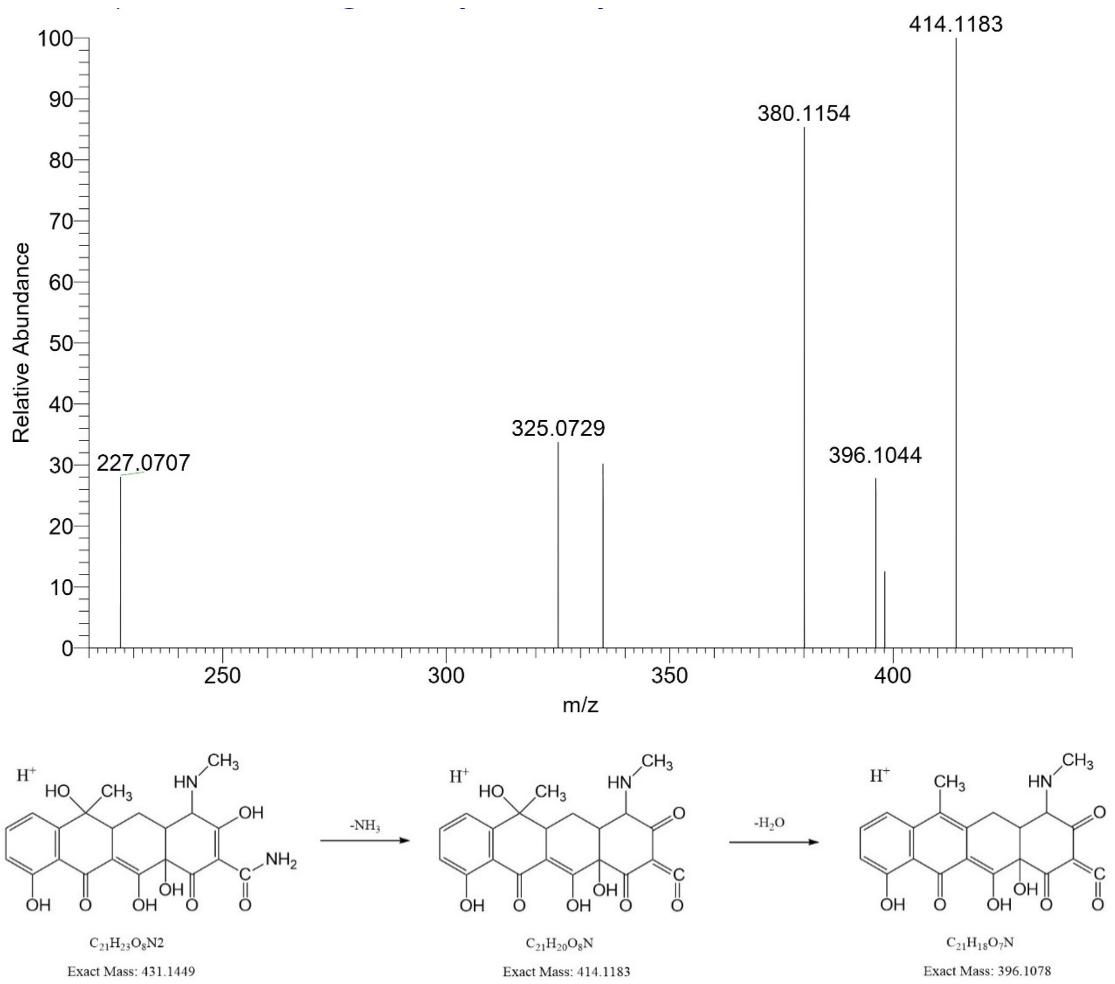

Figure S18. The MS2 fragmentation profile and proposed fragmentation pattern of TP431.

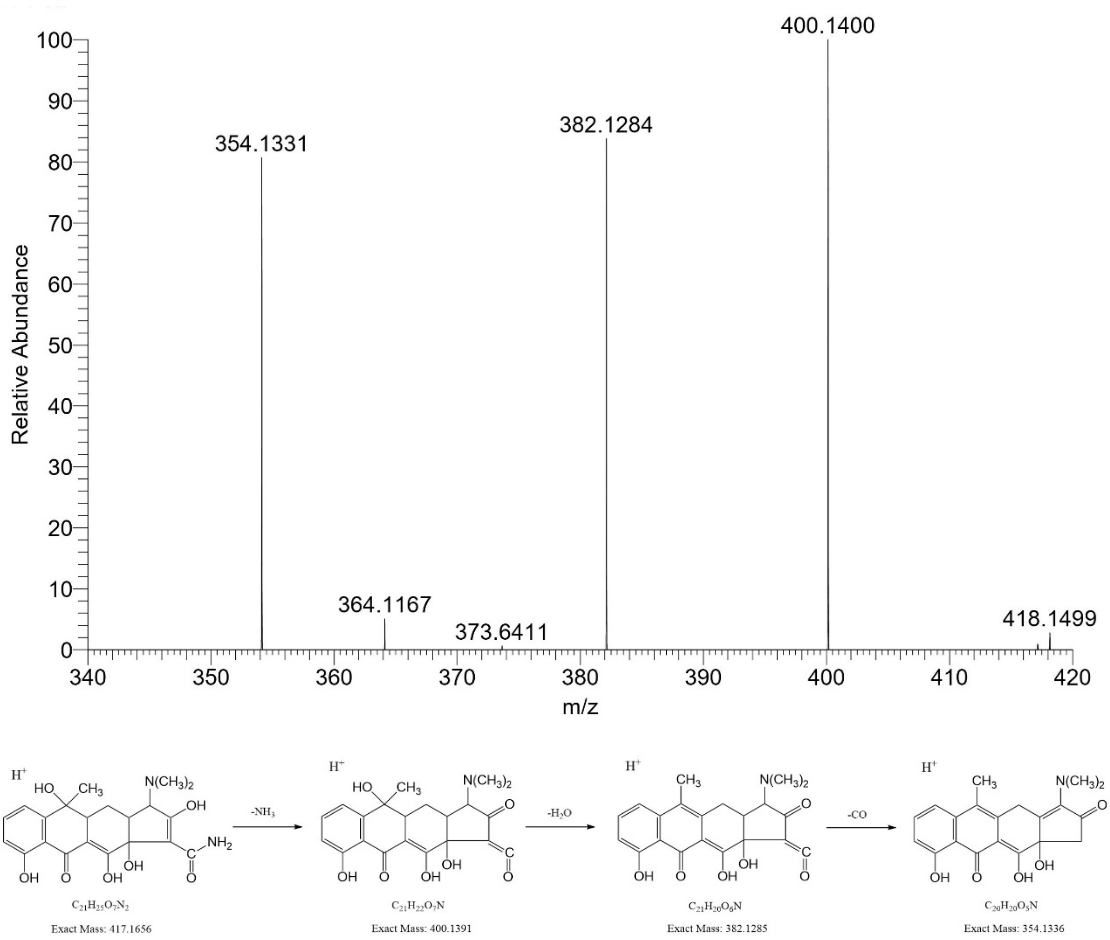

Figure S19. The MS2 fragmentation profile and proposed fragmentation pattern of TP417.

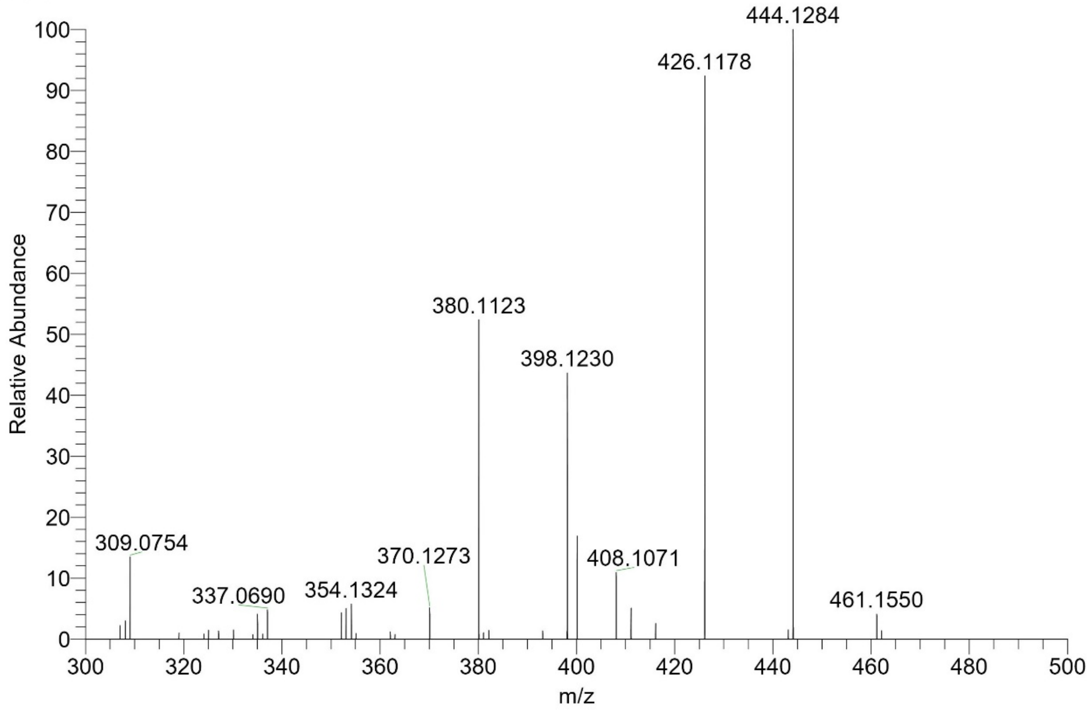

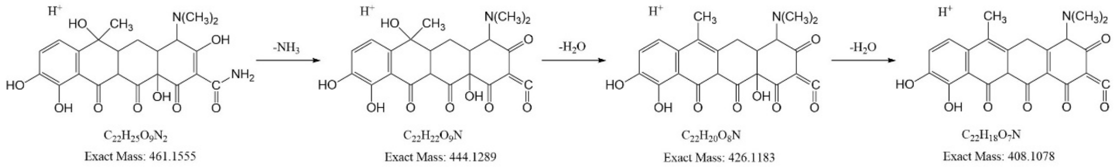

Figure S20. The MS2 fragmentation profile and proposed fragmentation pattern of TP461.

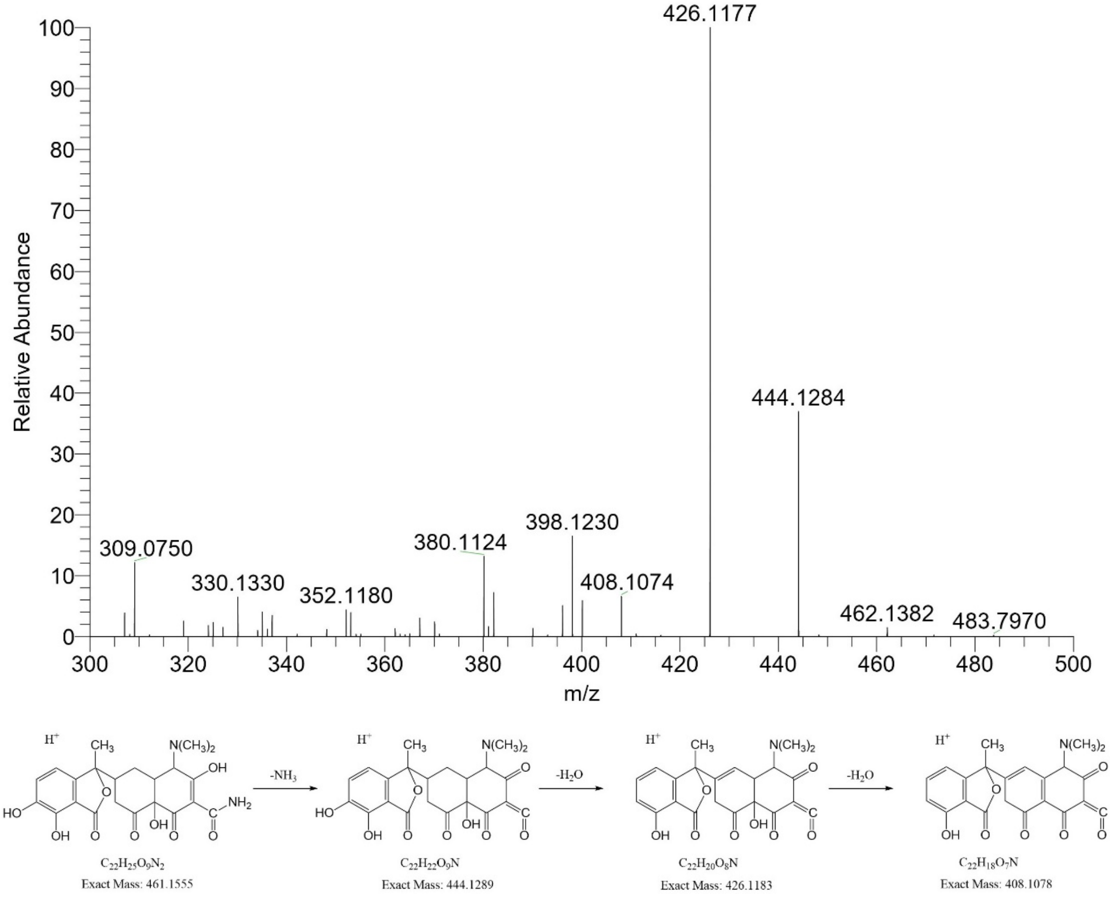

Figure S21. The MS2 fragmentation profile and proposed fragmentation pattern of ISO-TP461.

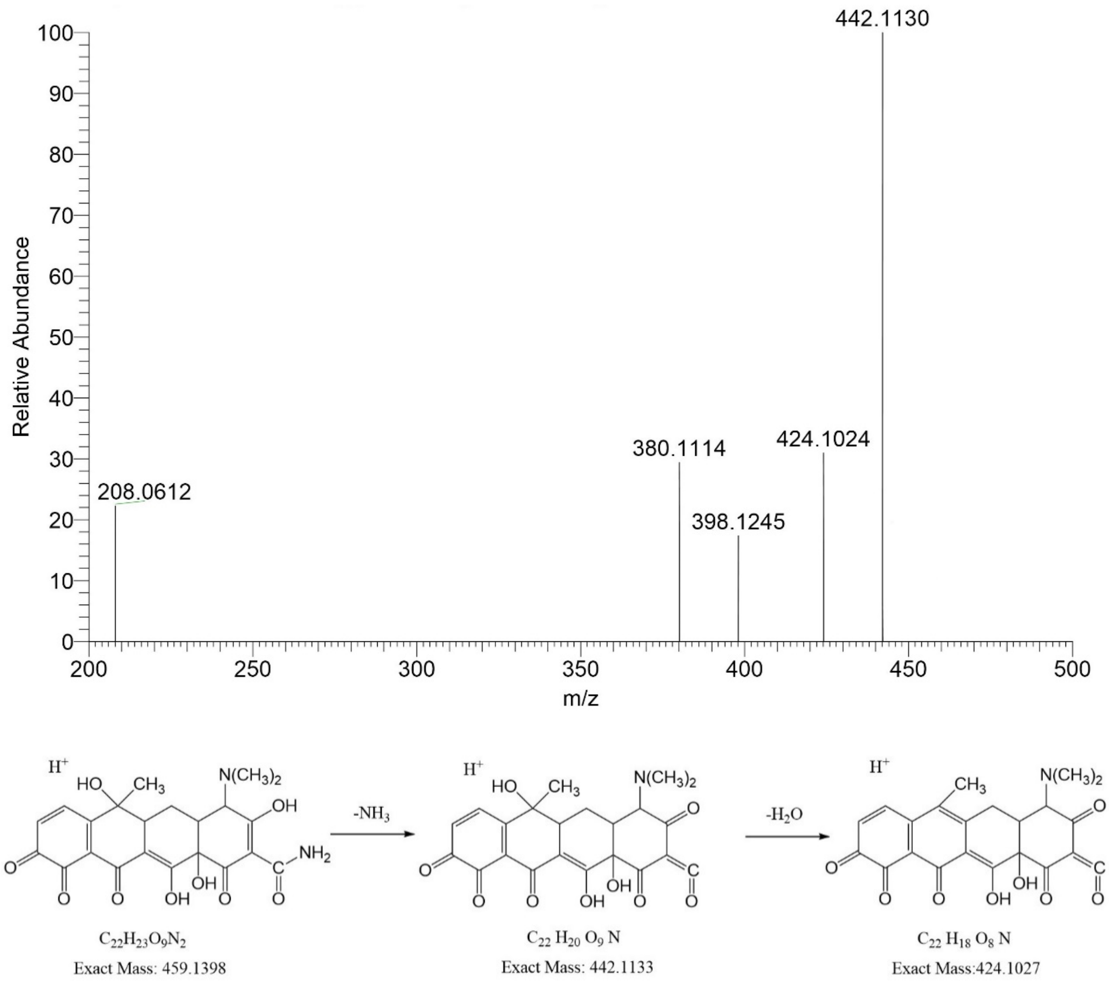

Figure S22. The MS2 fragmentation profile and proposed fragmentation pattern of TP459.

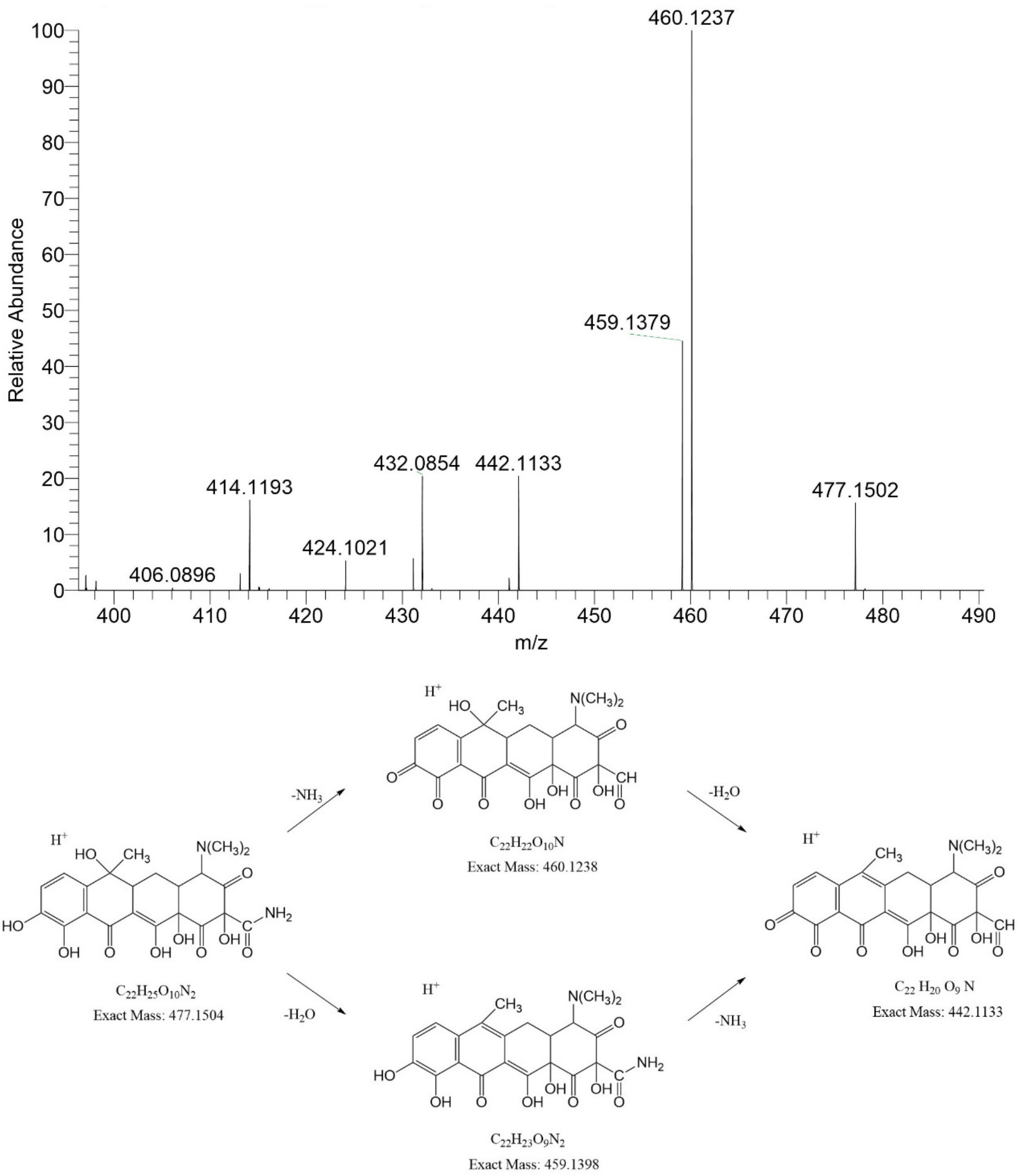

Figure S23. The MS2 fragmentation profile and proposed fragmentation pattern of TP477.

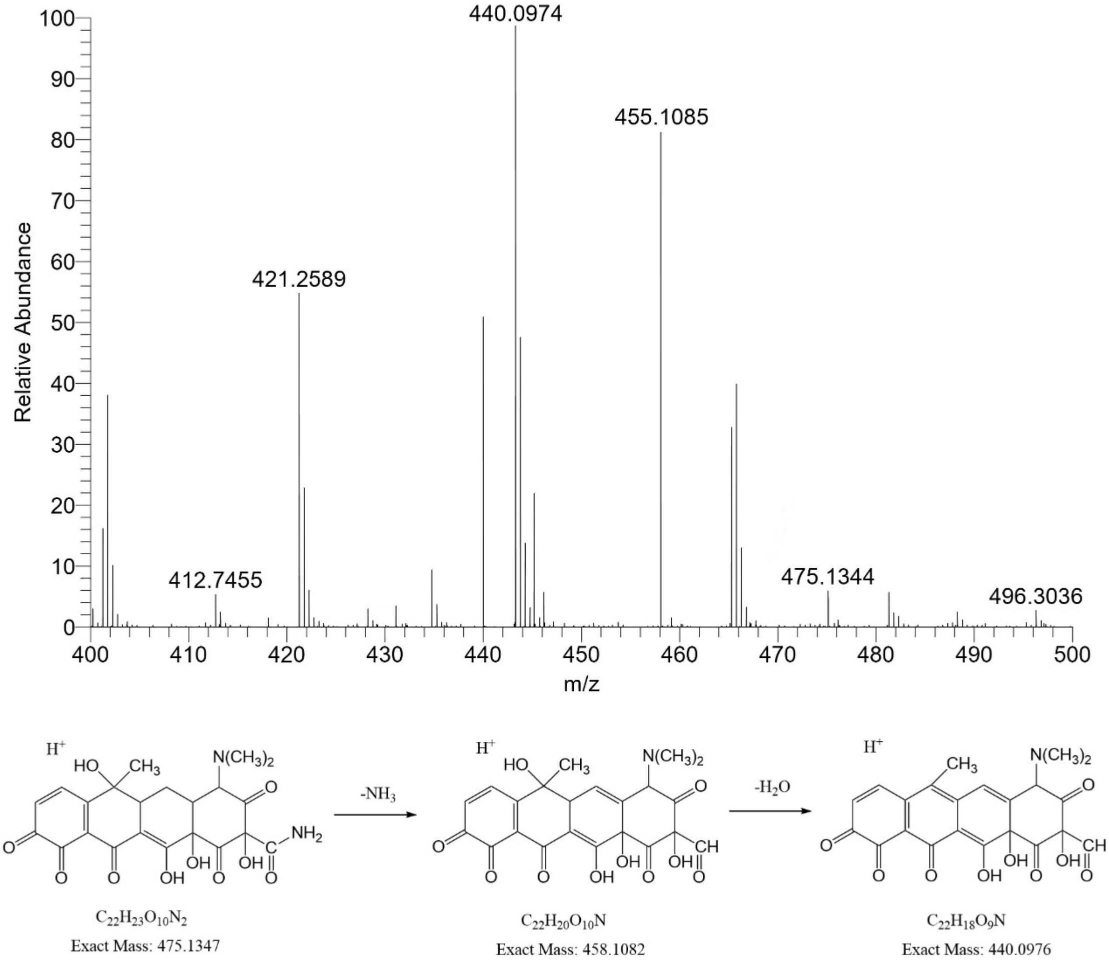

Figure S24. The MS2 fragmentation profile and proposed fragmentation pattern of TP475.

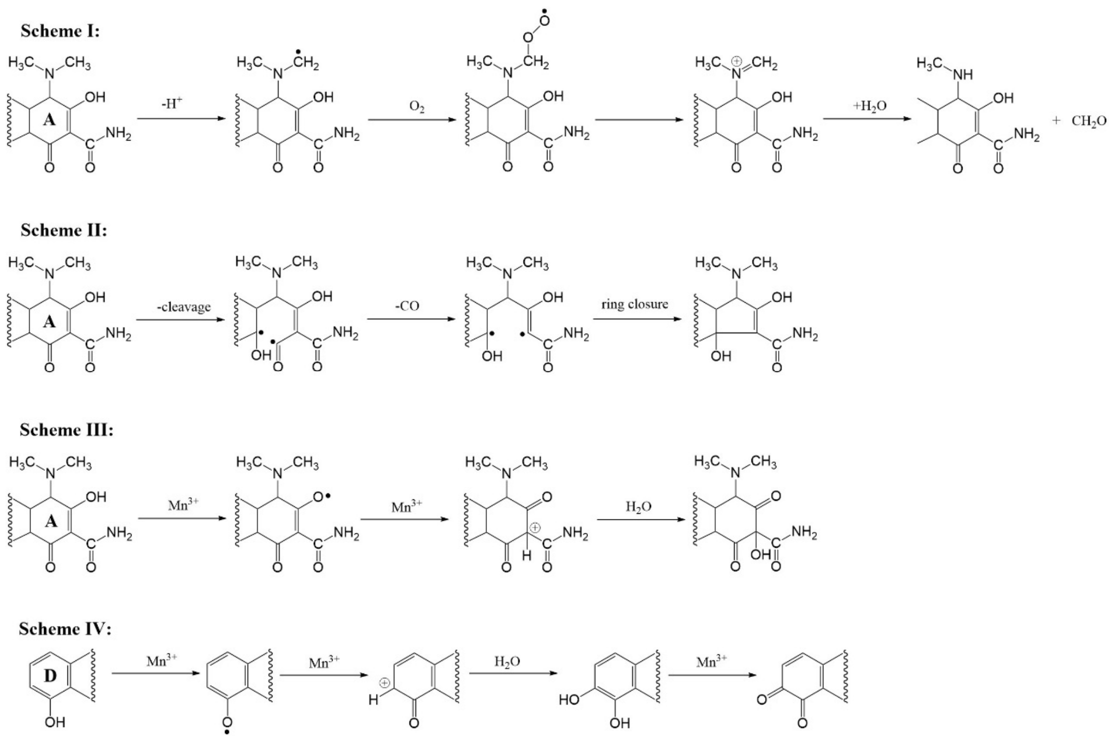

Figure. S25. Mechanistic steps Scheme I Scheme II Scheme III Scheme IV.

Table.S1 Factors and corresponding levels in L_8 (2^3) orthogonal experimental design.

level	Glucose/$g \cdot L^{-1}$ (X1)	Ammonium tartrate/ $g \cdot L^{-1}$ (X2)	$MnSO_4$/$mmol \cdot L^{-1}$ (X3)
1	10	1.62	1.00
2	2	0.20	0.02

Table.S2 Matrix layout of the L_8 (2^3) orthogonal array design.

Experiment No.	Glucose ($g \cdot L^{-1}$)	Ammonium tartrate ($g \cdot L^{-1}$)	$MnSO_4$ ($mmol \cdot L^{-1}$)
1	2	1.62	0.02
2	10	1.62	0.02
3	2	0.20	0.02
4	10	0.20	0.02
5	2	0.20	1.00
6	10	0.20	1.00
7	10	1.62	1.00
8	2	1.62	1.00

Reference:

Rahul, D., Aditi, K., Divyashri, B., Ali, M., Amitava, M., Ram, M., Pavel, F., 2017. Enzymatic Degradation of Lignin in Soil: A Review. Sustainability 9, 1163.

Tien, M., Kirk, T.K., 1988. Lignin peroxidase of Phanerochaete chrysosporium. Methods in Enzymology. Academic Press, pp. 238-249.
